# Convolutional neural networks for the diagnosis and prognosis of the coronavirus disease pandemic

**DOI:** 10.1186/s42492-021-00078-w

**Published:** 2021-05-05

**Authors:** Sneha Kugunavar, C. J. Prabhakar

**Affiliations:** Department of Computer Science, Kuvempu University, Shimoga, Karnataka 577451 India

**Keywords:** COVID-19, Neural network, Convolutional neural network, Deep learning, Medical image analysis

## Abstract

A neural network is one of the current trends in deep learning, which is increasingly gaining attention owing to its contribution in transforming the different facets of human life. It also paves a way to approach the current crisis caused by the coronavirus disease (COVID-19) from all scientific directions. Convolutional neural network (CNN), a type of neural network, is extensively applied in the medical field, and is particularly useful in the current COVID-19 pandemic. In this article, we present the application of CNNs for the diagnosis and prognosis of COVID-19 using X-ray and computed tomography (CT) images of COVID-19 patients. The CNN models discussed in this review were mainly developed for the detection, classification, and segmentation of COVID-19 images. The base models used for detection and classification were AlexNet, Visual Geometry Group Network with 16 layers, residual network, DensNet, GoogLeNet, MobileNet, Inception, and extreme Inception. U-Net and voxel-based broad learning network were used for segmentation. Even with limited datasets, these methods proved to be beneficial for efficiently identifying the occurrence of COVID-19. To further validate these observations, we conducted an experimental study using a simple CNN framework for the binary classification of COVID-19 CT images. We achieved an accuracy of 93% with an F1-score of 0.93. Thus, with the availability of improved medical image datasets, it is evident that CNNs are very useful for the efficient diagnosis and prognosis of COVID-19.

## Introduction

Coronavirus disease (COVID-19), caused by the severe acute respiratory syndrome coronavirus-2, is a pathogenic viral infection [1]. The spread of the COVID-19 pandemic has become an issue of global concern [2], which has forced the World Health Organization to reassess the conventions of the healthcare system [3]. The rapid spread of COVID-19 has also hampered the supply chain of critical care equipment and medical products [1, 4–6]. The current crises has attracted the attention of academicians, researchers, and scientists from different backgrounds [7–11]. New technologies are being developed to provide intelligent solutions for complex treatments and procedures, leading to an industrial revolution [12–14]. Doctors, virologists, disease transmission experts, and phylogeneticists have teamed up with policymakers to incorporate the available knowledge regarding the disease pathogenesis and control the spread of the infection. Effective screening of patients is the fundamental step in fighting COVID-19 so that infected patients can be isolated and treated. Laboratory diagnosis through reverse transcription-polymerase chain reaction (RT-PCR) is considered as the reference standard for the screening of COVID-19. However, false-negative instances of the RT-PCR tests have been progressively reported [15]. Laboratory findings through RT-PCR testing alone cannot be used as a substantial basis to confirm COVID-19 diagnosis. Other diagnostic methods, along with clinical correlations, are essential to confirm the infection status. Medical imaging is being tested as a potential screening tool for the early diagnosis and prognosis of COVID-19 [16]. Studies suggest that medical imaging will perform a crucial role in validating RT-PCR tests [17].

The interpretation of medical images is by default performed by experts in the field, such as radiologists and physicians. As medical data vary significantly from one patient to another depending on the disease, the task of diagnosis becomes labor intensive. Further, the interpretation is highly dependent on clinicians who exhibit limitations in terms of experience, speed, and fatigue. With the current crisis, the ability of the healthcare system to cope with the pandemic has been tested. One way to achieve this is to conduct medical image analysis through an automated, precise, and efficient computational framework such as a neural network, which is capable of replicating the accuracies of a trained human brain. In fact, the quantum of medical image analysis that an efficient neural network can perform at any given time is significantly greater than that of a human cortex.

The application of neural networks to tackle COVID-19 is primarily achieved by providing meaningful insights to medical image data [18]. Driven by a combination of factors such as public health emergencies, availability of a large collection of data, and advances in technology, several neural network models have been constructed [19]. A convolutional neural network (CNN) is a class of deep neural networks that are primarily employed for medical image processing. These neural network models help extract specific findings from chest radiology images of COVID-19 patients. In this article, we discuss different types of CNN models that have been proposed to recognize the patterns in chest X-ray and computed tomography (CT) images of COVID-19 patients, enabling automatic detection, segmentation, and classification of images. Keywords such as COVID-19, RT-PCR, CT, X-ray, neural network, CNN, deep learning, and medical image analysis were used to search for articles through the websites of PubMed, Radiopaedia, and Google Scholar. Further, to gain an intuitive and simplified understanding of the CNNs for COVID-19 image classification, we conducted an experimental study using a simple CNN framework. The experiment was conducted to classify COVID-19 and non-COVID-19 CT images using a publicly available dataset.

## Overview of the CNN

This section provides a formal introduction to the neural network approach and architecture. A neural network is a learning algorithm and is the primary element of most deep learning methods. Neural networks or artificial neural networks are capable of functioning like the human cerebrum. These networks can achieve faster and more reliable pattern recognition and aid in interpreting medical images. The neural network is an information processing paradigm designed to mimic the processes of the human brain and analyze the available information.

**Fig. 1:**
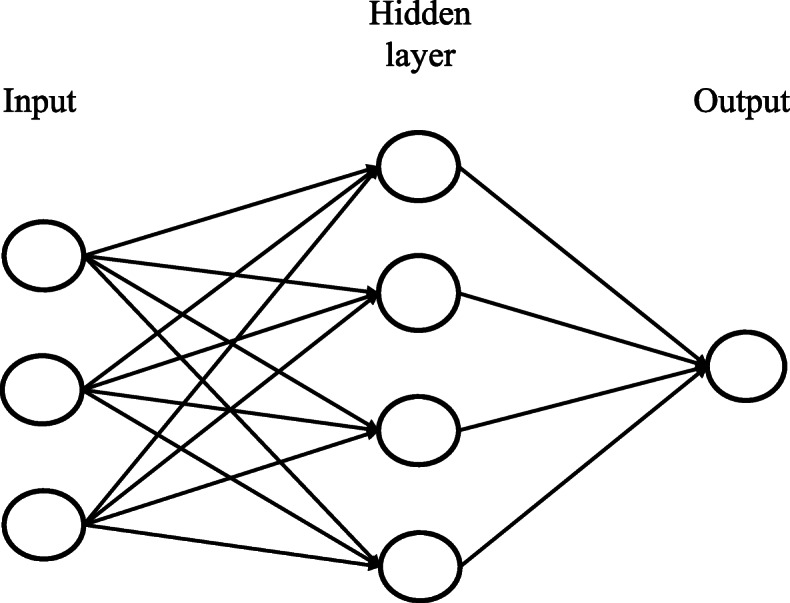
Simple neural network architecture

The neural network architecture consists of neurons along with the input layer, output layer, and one or more intermediate layers called hidden layers. Neurons are mathematical operations. The layers of neural networks are highly interconnected and coordinate in a distributed way to learn from the given input information and optimize its final output. Figure 1 shows a simple neural network architecture. First, the data enter the network through the input layer, followed by multiple hidden layers, where the data are transformed as they pass through each layer. Further, the resulting data from the previous layer are passed to the output layer, which produces the desired result. Neural networks that involve multiple hidden layers are considered as deep neural networks, resulting in the introduction of the term “deep learning” [20].

Deep learning techniques can be easily adapted for different applications and domains when compared to classical machine learning methods. Unlike traditional systems that use handcrafted features [21], these deep neural network models learn features from the data, i.e., they can completely discover the morphological patterns in an image from the data. The neural network technique does not require manual preprocessing of the raw data. The different types of neural networks include CNNs, recurrent neural networks, long short-term memory networks, and generative adversarial networks (GANs). The most popular model considered for medical image analysis is the CNN, also known as ConvNet.

**Fig. 2:**
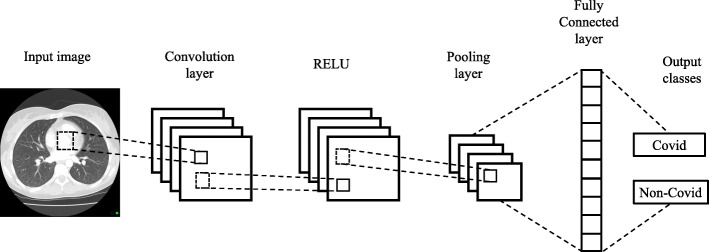
Basic CNN architecture for image classification

The architecture of a CNN can help achieve multilevel hierarchical feature learning. The early layers of CNN can fetch the low-level features (texture and shape), while the deep layers can extract the high-level semantic features that are combined to locate the key points accurately. Because the input consists of an image, the CNN compels the architecture more sensibly. The ConvNet layers have neurons arranged in three dimensions, that is height, width, and depth. Figure 2 shows the basic CNN architecture used for image classification. The CNN architecture includes an input layer, convolutional layer, rectified linear unit (ReLu), pooling layer, fully connected layer, and output layer. These layers are stacked to build a full CNN architecture. Feature extraction is performed through the convolutional, ReLU, and pooling layers. The classification is performed in the fully connected layer. Recognizing the layers and their connectivity in a deep neural network is more complex than running a pretrained general architecture. Designing an end-to-end neural network model can improve the recognition of the relationship between layers. As it filters irrelevant features, the model becomes more sensitive to meaningful patterns and handles the task in a more significant way.

The key research areas and applications of neural networks in medical image analysis include classification, detection, segmentation, localization, and registration. The neural network can provide a method to augment the early detection of COVID-19. Transfer learning has facilitated the utilization of pretrained neural network models for processing, analysis, segmentation, and classification of radiology images of COVID-19 patients. Further, certain neural networks have been further expanded in accordance with the task to be accomplished.

## Medical image analysis of the COVID-19

**Fig. 3:**
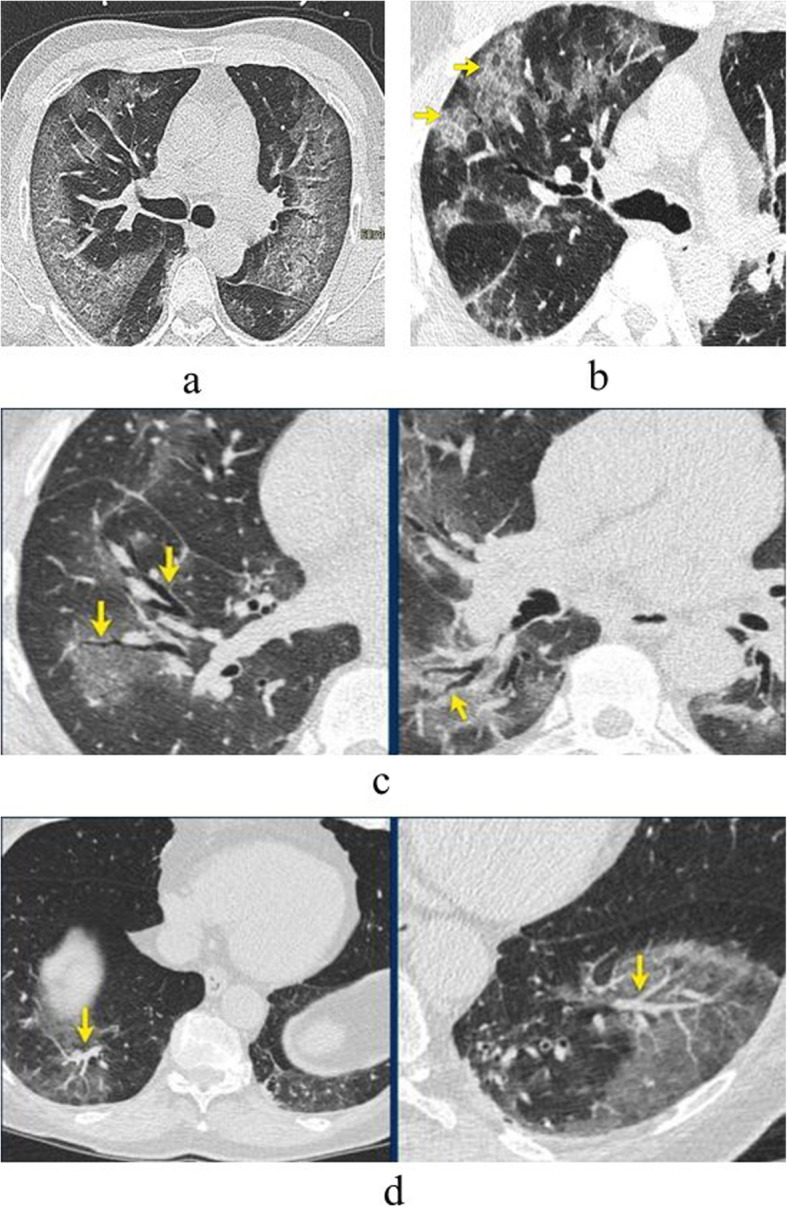
Common image findings observed in lung CT scans of the patients infected with COVID-19. **a** Ground-glass opacity; **b** Crazy paving; **c** Traction bronchiectasis; **d** Vascular dilation [26]

**Fig. 4:**
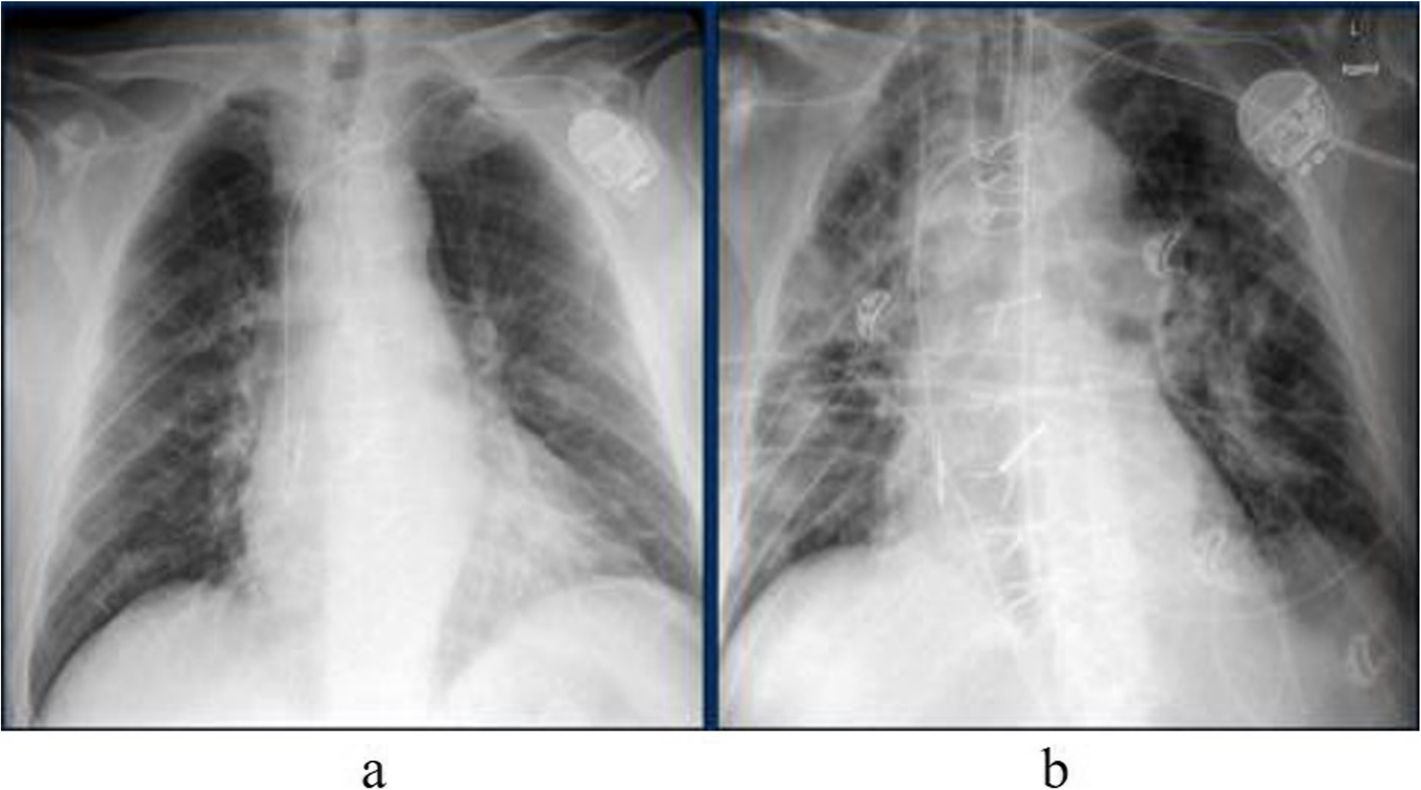
X-rays of a patient with COVID-19. **a** Normal; **b** After 4 days with bilateral consolidations [26]

Effective screening is an important step in limiting the spread of COVID-19 and creating awareness so that people can self-isolate if they develop any symptoms. Laboratory diagnosis through RT-PCR is considered the standard screening method where the respiratory samples are tested, and the results are obtained within a few hours to two days. Several RT-PCR analyses were developed for the detection of COVID-19. However, because of suboptimal sensitivity, the RT-PCR tests may sometimes yield false-negative results. In one of the studies conducted [22], in the early stages with the first appearance of the symptoms, the CT scans of a few patients showed the presence of COVID-19 infection, whereas the RT-PCR test results of these patients were negative. Both tests were performed repeatedly for several days, and the RT-PCR results subsequently confirmed that the CT results were true. RT-PCR can yield a false-negative result if the viral load is below the detection limit in the early stages or if there is an error in sampling [23]. Radiological images, such as chest X-rays and CT scans, are considered as an alternative screening method for COVID-19. A large amount of research has been conducted to prove the convenience of thoracic radiology evaluation for the diagnosis of COVID-19. Research suggests that the primary findings of COVID-19 are pneumonia, as observed by examining the X-ray and CT images of the thorax. Early investigators discovered a few common imaging patterns in the X-ray and CT images [15, 24]. Depending on the time after the onset of the disease, image characteristics of the infected patients vary from normal to dispersed patchy consolidations. The image findings frequently observed in the CT scans of the infected patients were ground-glass opacities (GGOs), crazy-paving appearance, air-space consolidation, bronchovascular thickening, and traction bronchiectasis [25, 26]. Figure 3 shows these common image findings in lung CT images. Figure 4 shows the normal and affected X-ray images of COVID-19 patients [26].

Chest radiology imaging for diagnosis has several advantages over the conventional RT-PCR method. Methods involving radiology imaging tend to be quick and can simultaneously analyze multiple cases. Given the significance of radiography in the modern health care system, a radiology framework is available in all hospitals, which is more convenient and easily accessible. This framework is significantly helpful in hospitals without any testing kits or with only a limited number of kits. Further, not all hospitals have the required laboratory setup to assist in a high rate of sample testing procedures. The imaging method appears to be a valuable tool for COVID-19 clinical management and therefore can be used for the diagnosis, detection, follow-up, and evaluation of the severity of the infection.

To conduct any research, datasets are significantly essential. Some of the COVID-19 image datasets were made publicly available to encourage institutes and organizations to conduct research and discover solutions. Table 1 lists datasets consisting of chest X-ray and CT images of COVID-19 patients.

**Table 1:** List of COVID-19 datasets available in the public domain

Dataset	Modality	Link
COVID-CT [27]	CT	https://github.com/UCSD-AI4H/COVID-CT
Eurorad [28]	X-ray and CT scans	https://www.eurorad.org/advanced-search?search=COVID
BSTI [29]	CT and CR	https://www.bsti.org.uk/covid-19-resources/
COVID-chest X-ray [30]	X-ray	https://github.com/ieee8023/covid-chestxray-dataset
COVID-19 CT segmentation data [31]	CT	http://medicalsegmentation.com/covid19/
SIRM [32]	X-ray and CT images	https://www.sirm.org/category/senza-categoria/covid-19/
Actualmed COVID-19 chest X-ray [33]	X-ray	https://github.com/agchung/Actualmed-COVID-chestxray-dataset
Kaggle [34]	X-ray and CT	https://www.kaggle.com/datasets?search=covid
Radiology assistant [26]	X-ray and CT	https://radiologyassistant.nl/chest/covid-19/covid19-imaging-findings
COVIDx [35]	X-ray	https://github.com/lindawangg/COVID-Net

## CNN for COVID-19 medical image analysis

As CNNs are being widely used in medical image analysis, in this section, we list the major developments in neural network technology to gain an insight on how these networks are being employed for the detection, classification, and segmentation of COVID-19. Following an experimental study to obtain a better understanding of the CNNs for COVID-19 image classification, we discuss the advantages and challenges of using these methods.

### Detection and classification

CNNs are the best frameworks for the detection and classification of COVID-19 as they are capable of identifying the patterns present in chest radiology images. The classification process extracts important features and inputs them to the deep layers and classifies the affected chest CTs and X-rays from other images. Table 2 lists the CNN models developed for the detection and classification of COVID-19.

**Table 2:** List of CNNs used for the detection and classification of COVID-19

Task	Image modality	Dataset	Approach	Reference
Detection	X-ray	Normal, non-COVID-19 pneumonia, COVID-19	COVID-Net, tailored deep CNN	Wang et al. [36]
Detection	X-ray	Normal, viral, bacterial, COVID-19	CovXNet, CNN with transfer learning	Mahmud et al. [37]
Detection	X-ray	COVID-19, non-COVID-19 (includes normal and other pneumonia)	CNN-tailored shallow architecture, 5-fold cross-validation	Mukherjee et al. [38]
Detection	X-ray	Normal, COVID-19	ResNet50, InceptionV3 and Inception-ResNetV2, 5-fold cross-validation	Narin et al. [39]
Detection	X-ray	Normal, COVID-19, bacterial pneumonia	Modality-specific transfer learning with CNN, iterative pruning, ensemble strategies	Rajaraman et al. [40]
Detection	X-ray	COVID-19, non-COVID-19 pneumonia	ResNet50, VGG16, small CNN, ensemble of three CNNs, 10-fold cross-validation	Hall et al. [41]
Detection	X-ray	Normal, pneumonia and COVID-19	MobilNetV2	Apostolopoulos and Mpesiana [42]
Detection	X-ray	Normal, pneumonia and COVID-19	Concatenation Xception and ResNet50V2	Rahimzadeh and Attar [43]
Detection	X-ray	Common pulmonary diseases, COVID-19	MobileNetV2	Apostolopoulos et al. [44]
Detection	X-ray, CT scans	COVID-19, bacterial pneumonia, viral pneumonia	AlexNet, VGG, SqueezeNet, GoogLeNet, MobileNet, ResNet and DenseNet	Rehman et al. [45]
Detection	CT scan	COVID-19 positive and negative	VGG16, InceptionV3, ResNet50, DenseNet121, DenseNet201, decision fusion	Mishra et al. [46]
Classification	X-ray	Normal, COVID-19, pneumonia	ResNet152, SMOTE algorithm	Kumar et al. [47]
Classification	X-ray	Normal, COVID-19	COVIDX-Net, deep learning classifiers VGG16 and DenseNet201 showed good performance	Hemdan et al. [48]
Classification	X-ray	Normal, SARS, COVID-19	DeTraC, transfer learning, ResNet18	Abbas et al. [49]
Classification	X-ray	Normal, COVID-19, bacterial and viral pneumonia	Transfer learning with CNN	Ozturk et al. [50]
Classification	CT scan	Image patches of COVID-19 findings, COVID-19 and no-finding	VGG16, GoogleNet, and ResNet50, feature fusion and ranking technique, SVM classifier	Özkaya et al. [51]
Classification	CT scans	Viral pneumonia, COVID-19	M-Inception, transfer learning	Wang et al. [52]
Detection	X-ray	COVID-19, normal	CovidGAN, VGG16, GAN	Waheed et al. [53]
Detection	X-ray	COVID-19, normal	AlexNet, ResNet18, SqueezeNet, GoogLeNet, GAN	Khalifa et al. [54]
Classification	X-ray	COVID-19, normal, other pneumonia	VGG16, ResNet50, EfficientNetB0, GAN	Zebin and Rezvy [55]

Abbas et al. [49] developed a CNN that relies on a class decomposition approach called decompose, transfer, and compose (DeTraC) for classifying the chest X-rays of COVID-19 patients. DeTraC can manage any irregularities in an image dataset by examining class boundaries through the class decomposition mechanism. DeTraC showed an accuracy of 85.12% in detecting COVID-19 X-ray images from normal and severe acute respiratory syndrome cases. COVID-Net, a deep CNN model, was developed by Wang et al. [36]. The model achieved 93.3% accuracy while identifying the normal, COVID-19, and pneumonia-infected X-rays of patients. Hemdan et al. [48] proposed COVIDX-Net using X-ray images to identify the positive or negative status of COVID-19 infection in patients. COVIDX-Net involves seven deep learning classifiers, namely DenseNet201, Visual Geometry Group Network with 19-layered CNN (VGG19), InceptionV3, residual network V2 (ResNet-V2), Inception-ResNet-V2, extreme Inception (Xception), and MobileNetV2. VGG19 and DensNet201 showed the highest performance scores. The high computational speed of MobileNetV2 presents a scope for improvement; consequently, it can be integrated with smart devices.

A deep-transfer-learning-based approach was adapted by Narin et al. [39] using chest X-ray radiographs to predict normal and COVID-19 infected patients automatically. The pretrained models were tested for accuracy, where the ResNet50 model showed 98% accuracy, while InceptionV3 and Inception-ResNet-V2 achieved accuracies of 97% and 87%, respectively. Mahmud et al. [37] developed a deep CNN architecture called CovXNet, which uses depthwise convolution with varying dilation rates for extracting diversified features from chest X-rays. Different forms of CovXNets, which are highly scalable with a large receptive capacity, that can be employed to identify COVID-19 patients were designed. The abnormal regions of the X-ray images involving different types of pneumonia were identified by integrating gradient-based discriminative localization. By using the DarkNet model classifier as the basis of the you only look once object detection system, Ozturk et al. [50] developed a model to detect and classify the COVID-19 cases from X-ray images. The classification results of this algorithm were evaluated for both binary and triple classes, which demonstrated accuracies of 98.08% and 87.02%, respectively. Another study by Apostolopoulos and Mpeisiana [42] deployed transfer learning with a CNN for the automatic detection of COVID-19 from X-ray images.

For the automatic detection of COVID-19 positive cases using X-ray images, Mukherjee et al. [38] developed a tailored shallow architecture that consists of only four layers. The experiment was conducted with a smaller X-ray dataset with 130 COVID and 51 non-COVID cases for which the false-positive rate was zero. To avoid possible bias, five-fold cross-validation was performed. The accuracy of this lightweight CNN was 96.92%. Hall et al. [41] fine-tuned a pretrained ResNet50 on chest X-rays of patients with COVID-19 and pneumonia using 10-fold cross-validation. The ensemble of ResNet50, VGG16, and a CNN classifier developed by them showed an overall accuracy of 89.2%. Rahimzadeh and Attar [43] concatenated Xception and ResNet-50-V2 networks to develop a model for the classification of X-ray images into normal, pneumonia, and COVID-19 cases. The model achieved an overall accuracy of 91.4%. Kumar et al. [47] used ResNet152 and a machine learning classifier for the classification of COVID-19 and pneumonia patients using X-rays. The intraclass variations of the datasets were balanced using the synthetic minority oversampling technique or SMOTE. The random forest classifier achieved an accuracy of 97.3% and the extreme gradient boosting classifier achieved an accuracy of 97.7%.

Wang et al. [52] built a transfer learning neural network by modifying the Inception network, which was referred to as M-Inception, to extract graphical features and provide a clinical diagnosis ahead of a pathogenic test. The model achieved an accuracy of 82.9% while classifying the CT images of COVID-19 cases. Apostolopoulos et al. [44] trained MobileNetV2 from scratch to investigate the extracted features for classifying the seven classes of pulmonary diseases, including COVID-19, using X-ray images. The proposed model provided an overall classification accuracy of 87.66% for the seven classes and a specificity of 99.42% for detecting COVID-19.

Mishra et al. [46] employed a decision-fusion-based approach that combined the predictions of each baseline model, that is, VGG16, InceptionV3, ResNet50, DenseNet121, and DenseNet201 for detection of COVID-19 from chest CT images. The proposed model achieved an average accuracy of 88.34%, which outperformed the performance of each individual model; moreover, DenseNet121 performed the best while considering the individual models. The COVID-19 infected region appears in different levels of gray in the CT image of the lungs, which poses difficulty in the analysis of the image. Özkaya et al. [51] used these differences in the gray levels to generate two datasets: subset-1 (16 × 16) and subset-2 (32 × 32) by obtaining random patches from the CT images of COVID-19 patients. The pretrained networks, i.e., VGG16, GoogleNet, and ResNet50, were employed for feature extraction. Feature fusion was performed to obtain high-dimensional features, and feature ranking was performed. The support vector machine classifier was trained for classification purposes.

### Segmentation

Segmentation can help to provide the quantitative information necessary to detect COVID-19, analyze the severity, and extract the region of interest. Only a few works have employed segmentation to enhance the performance of developed frameworks. Table 3 lists the CNN models used for COVID-19 image segmentation. Alom et al. [57] used an Inception Residual Recurrent CNN (IRRCNN) with a transfer learning approach for the detection of COVID-19. The model was evaluated for both chest X-ray and CT images, and a quantitative evaluation was performed to determine the severity of COVID-19. The IRRCNN model was used to classify COVID-19 cases. NABLA-3 was employed for the segmentation of the infected lung region. The accuracy of the proposed method was 84.67% and 98.78% for X-ray and CT images, respectively.

**Table 3:** List of CNNs used for COVID-19 image segmentation

Task	Image modality	Dataset	Approach	Reference
Segmentation	CT scans	COVID-19 CT with annotation, random chest CT scans	COVIDSegNet, encoder and decoder, Progressive Atrous Spatial Pyramid Pooling	Yan et al. [56]
Classification, segmentation	X-ray, CT scan	Normal, COVID-19	COVID_MTNet, transfer learning with Inception Residual Recurrent Neural Network, NABLA-N network model	Alom et al. [57]
Segmentation	CT scans	COVID-19	VB-Net	Shan et al. [58]
Segmentation	CT scans	COVID-19	U-Net	Chen et al. [59]
Segmentation	CT scans	COVID-19	Inf-Net, Semi-Inf-Net	Fan et al. [60]
Segmentation	CT scans	COVID-19	COPLE-Net	Wang et al. [61]
Segmentation	CT scans	COVID-19	U-Net, CNN	Voulodimos et al. [62]

Rajaraman et al. [40] used an iteratively pruned deep learning model to detect the presence of COVID-19 using chest X-ray images. The best-performing models were pruned to reduce the complexity and improve the memory efficiency. Modality-specific transfer learning was performed by retraining the customized CNN and ImageNet models. These models were further combined with different ensemble strategies to improve the classification. U-Net-based semantic segmentation was performed to fetch the important lung features and separate them from the background. Weighted averaging of the best-performing pruned models demonstrated superior performance while classifying the X-rays into normal, bacterial pneumonia, or COVID-19 viral pneumonia cases with an accuracy of 99.01%. Yan et al. [56] evaluated a three-dimensional deep learning model called COVID-SegNet. The model was used to enhance the boundary of the COVID-19 pneumonia-infected lung CT images through segmentation. Feature variation and atrous spatial pyramid pooling blocks were used to highlight the infected lung area and capture global information for semantic segmentation. The proposed method achieved a dice similarity coefficient of 0.987 and 0.726 for lung and COVID-19 segmentation, respectively.

To expedite the process of manual delineation of the infected region in CT images, Shan et al. [58] developed a framework. The voxel-based broad learning network (VB-Net) was employed for segmenting the COVID-19 infected region from lung CT images. The human-in-the-loop (HITL) strategy was adapted where the radiologists monitored the proposed deep learning model to improve the automatic annotation of each case. The dice similarity coefficient of the proposed model was 91.6% ± 10% between automatic and manual segmentation. Chen et al. [59] proposed a modified U-Net model. Here, an aggregated residual transformation was used to enhance the feature extraction, which was combined with the soft attention mechanism to obtain high-quality multiclass segmentation of the infected region from the COVID-19 chest CT images.

Fan et al. [60] developed a COVID-19 lung infection segmentation deep network called Inf-Net to automatically delineate the infected region from CT images. A parallel partial decoder was used to extract the high-level features and produce a global map. To improve segmentation, implicit reverse attention and explicit edge attention were used to enhance the boundaries. Wang et al. [61] developed a noise-robust framework called COVID-19 pneumonia lesion segmentation network (COPLE-Net) to read noisy labeled CT images. The noise-robust dice loss and COPLE-Net were fused with an adaptive self-ensemble framework for training. Images were grouped based on the size of the lesion. COPLE-Net outperformed all other models when dealing with different lesion sizes.

### Data augmentation using GAN

Smaller datasets are considered to achieve satisfactory accuracy rates. However, as the amount of data increases, the results are correspondingly improved. At the beginning of the pandemic, the amount of data available was insufficient to add more variability to the dataset. Hence, synthetic data augmentation was performed using the GAN. Waheed et al. [53] proposed an auxiliary classifier GAN called CovidGAN by generating synthetic chest X-ray images. VGG16 was used as the base network. The aim was to prove the advantage of using synthetic images produced from CovidGAN to enhance the performance of CNNs for COVID-19 diagnosis. Because of the synthetic images, the accuracy of the CNN increased from 85% to 95%.

Khalifa et al. [54] fine-tuned CNN by deep transfer learning along with GAN for the detection of COVID-19 using X-ray images. The models for deep transfer learning included AlexNet, GoogLeNet, SqueezeNet, and ResNet18. The research used only 10% of the dataset for training and generated 90% of images using GAN to prove the efficiency of the proposed model. With GAN as the image augmenter, ResNet18 outperformed the other models by showing 99% accuracy. Zebin and Rezvy [55] employed pretrained CNNs VGG16, ResNet50, and EfficientNetB0 for the classification of COVID-19, pneumonia, and normal X-ray images. They also employed CycleGAN for data augmentation. The overall accuracies achieved were 90%, 94.3%, and 96.8% for VGG16, ResNet50, and EfficientNetB0, respectively.

## Experimental study

We attempted to gain an intuitive and simplified understanding of the CNNs for COVID-19 image classification. Consequently, we conducted a small-scale experiment using a simple CNN framework as a binary classifier for classifying the CT images into COVID-19 and non-COVID-19 classes [63]. The CNN framework was fine-tuned according to our requirements.

### Dataset

**Fig. 5:**
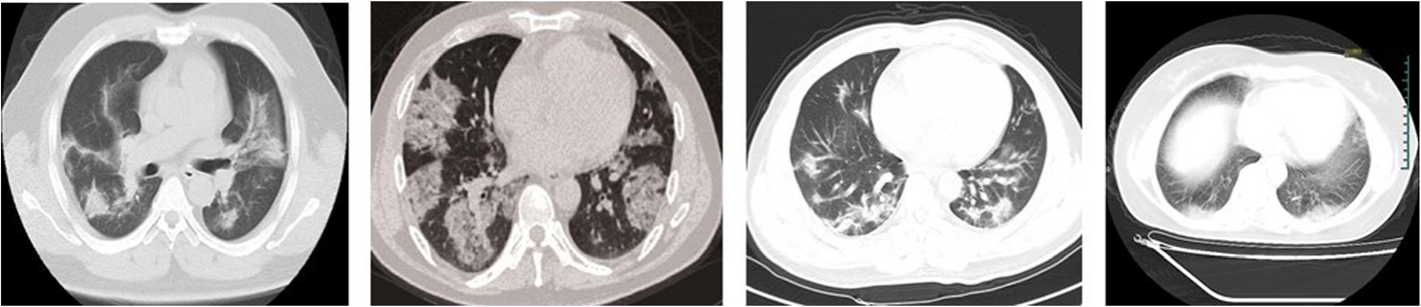
Sample COVID-19 CT images from the COVID-CT dataset

The publicly available COVID-CT dataset was used [64]. This dataset was downloaded from Kaggle [27]. The practicality of this dataset was confirmed by radiologists. The COVID-CT dataset consists of 349 COVID-19 CT images from 216 patients and 397 non-COVID-19 CT images. Figure 5 shows the sample COVID-19 affected CT images from the COVID-CT dataset. By understanding the importance of images and labels for training and testing of the CNN classifier, the dataset was labeled as COVID and non-COVID images, which were later transformed into arrays.

### Experimental setup

The CNN framework used consisted of convolutional, pooling, and fully connected layers. The convolutional and pooling layers perform feature extraction, whereas the fully connected layer maps the extracted features into the output, which, in this case, was classification. The CT images were resized to 200 × 200 pixels. A four-layered CNN with an Adam optimizer was employed to optimize the weights of the model. The learning rate was set to 0.001, the epoch value was three, and the softmax activation function was used. As CNN cannot directly work with categorical data, the images and labels are transformed into an array using one-hot encoder. Then, the dataset was split into a training set to train the network and a validation set for cross-validation of the model during the training phase. The validation set monitored the model performance based on which the parameters were fine-tuned, and a final model was selected. The CNN framework was implemented using Python, Keras with Tensorflow, and a graphical processing unit on an Intel Core i5 processor.

### Performance analysis

To evaluate the performance of the proposed CNN model we used the confusion matrix, which gives a holistic view of how well the model is performing (Table 4). Using the confusion matrix, the precision, recall, F1-score, and accuracy values were computed as follows.

1 $$ Precision=\frac{TP}{TP+ FP} $$

2 $$ Recall=\frac{TP}{TP+ FN} $$

3 $$ F1- score=\frac{2\times P\times R}{P+R} $$

4 $$ Accuracy=\frac{TP+ TN}{TP+ TN+ FP+ FN} $$

**Table 4:** Confusion matrix

	Predicted COVID-19	Predicted non-COVID-19
Actual COVID-19	True positive	False negative
Actual non-COVID-19	False positive	True negative

### Results

**Fig. 6:**
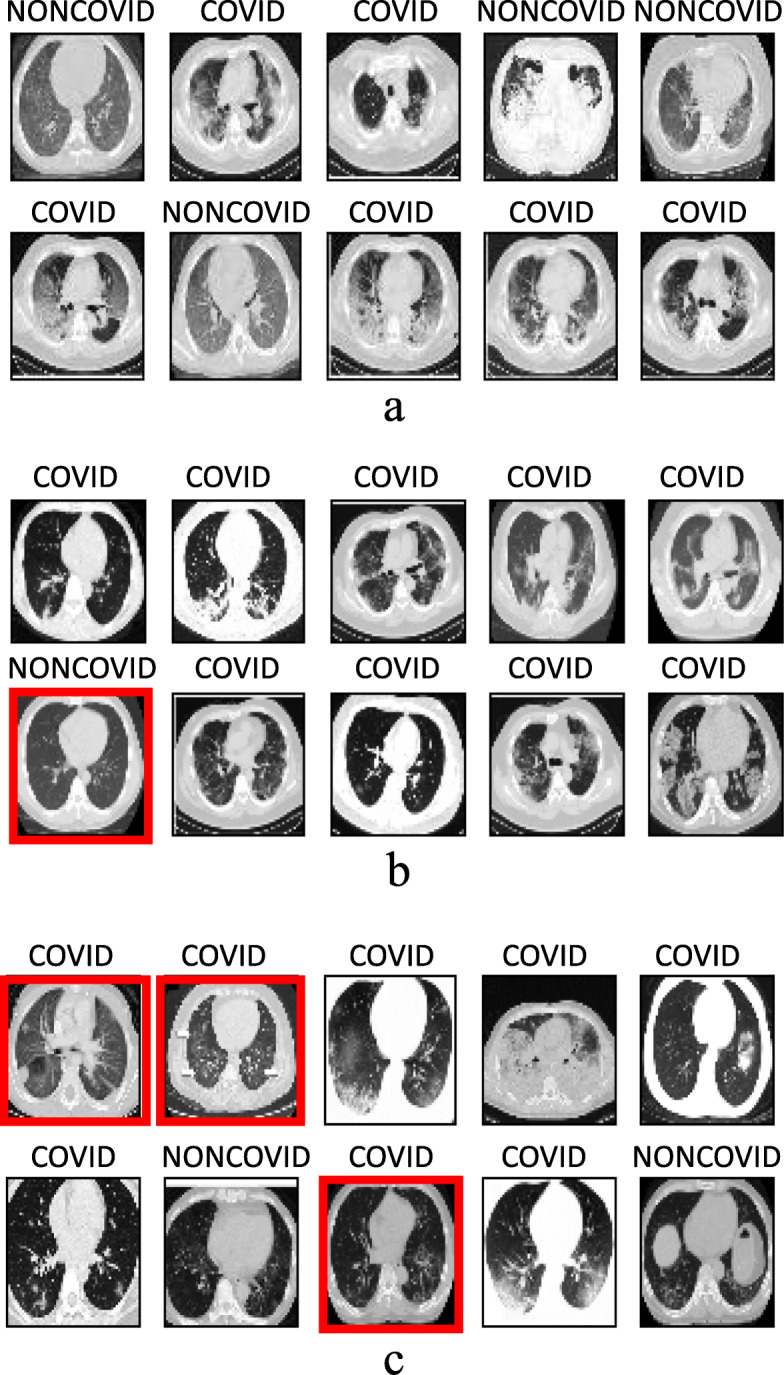
Classification performed by CNN architecture in the experimental study

To obtain a suitable result and for significant evaluation of the final model, the CNN was fed with a testing dataset consisting of 60 unlabeled, unseen COVID and non-COVID CT images. We used a straightforward approach without data augmentation. The classification results of the final CNN model are shown in Fig. 6. CT images that were wrongly classified are marked by a red square, as shown in Fig. 6b and c. We evaluated the performance of the classification model using the confusion matrix method. The above CNN model for the classification of COVID-19 CT images yielded an accuracy of 93% with a precision value of 0.91, a recall value of 0.96, and an F1-score of 0.93. This experiment was conducted to obtain a better understanding of the application of CNNs for COVID-19 image classification. The CNN model classifies the images based on valid information that is available in the lung images by ignoring the artifacts and false visual indicators. However, the model can be further improved by adding more CT images to the dataset, which can yield better results. Further, the CNN model should be examined by clinical experts to create a production-ready solution. The next section provides an overview of the protocols to be followed to design a CNN that can satisfy the clinical requirements.

## Discussion

In this section, the advantages and challenges of the aforementioned different studies are discussed. Tables 2 and 3 list various CNN frameworks used for the detection, classification, and segmentation of COVID-19 radiology images. It can be noted from the tables that CNNs are most commonly used for automated detection and classification. While analyzing the modalities used for the experiment, chest X-rays are mainly used for detection, whereas CT scans are used for classification. The segmentation of COVID-19 findings is predominantly performed using CT scans. The models listed in Table 2 either perform binary classification, i.e., normal versus COVID-19, or triple class classification, i.e., COVID-19 versus normal versus bacterial or viral pneumonia.

Researchers have used transfer learning to accelerate the learning process and reduce the requirement for large training data by employing pretrained CNN models. These CNN models were trained on a dataset that included COVID-19 medical images of the patients. Here, the early layers of the CNN learned basic features, and the high-level features were learned by the fine-tuned upper layers. The base models of most of the proposed CNNs are AlexNet, VGG16, ResNet, DensNet, GoogLeNet, MobileNet, Inception, and Xception (Table 2). If we consider the evaluation matrix, many of the proposed models show promising results.

The given images of the training dataset act as visual cues that include a variety of features. Deep neural networks learn by adjusting the weights that are backpropagated. This requires the model to learn from a large dataset covering all the possibilities for obtaining the highest accuracy when compared to state-of-the-art methods. The lack of publicly available datasets hinders the use of large medical images for training. Most of the models were trained and tested on a small dataset. The studies conducted involved images of COVID-19, bacterial, and other viral pneumonia. However, the factors such as age, sex, and other primary health conditions, such as diabetes, hypertension, and cardiovascular diseases, were not considered, which does not create a well-defined dataset and might not yield good results.

A large clinical dataset is used in the CNN to improve generalizability and limit overfitting. Although few techniques have encouraged learning on small datasets, as depicted in the articles, a well-annotated large medical dataset is still required. The vast majority of the eminent achievements of CNNs are typically based on a large amount of data. Unfortunately, building such datasets in medicine is expensive and requires a large amount of work by specialists; moreover, ethical and privacy issues must also be considered.

Another challenge due to the limited accessibility of medical datasets is the classification of medical images for clinical diagnosis. Because of the commonality that exists between COVID-19 and other types of bacterial or viral pneumonia, it is difficult to focus on the contrast between them. Most of the experiments performed by the authors did not explicitly confirm the recognition of COVID-19 and other pneumonia from identical CT findings. Further, there is a contradiction in the suitability of the modalities. In a few of the early instances, the CT images of patients with mild or no symptoms posed a difficulty in early detection. This limits the use of CT scans in testing. Considering the cost, CT scans are also more expensive, and exposure to radiation is higher than that of X-rays. However, CT scans are more detailed than X-ray scans.

To overcome the issue of the database, few authors used GAN models for data augmentation. Even though no new information is added to the network, to compensate for the inefficiency of the training dataset, existing image data were augmented, which increased the size of the dataset. One advantage of data augmentation is that it can also make the model more robust to overfitting.

Pretrained networks can add bias to the network, often leading to a local minimum and suboptimal solution. Developing an efficient and robust computer-aided diagnosis system for the diagnosis of COVID-19 is a difficult task. These pretrained models learn from the ImageNet database, which is different from the radiology images.

Most of the CNN frameworks that are listed in Table 2 focus on detection and classification, whereas the CNN frameworks for segmentation that are listed in Table 3 are comparatively lesser in number. U-Net and VB-Net were used for the segmentation of the lesion area (Table 3). These frameworks have demonstrated good results. However, when the effectiveness of segmentation is evaluated, there is no standard well-annotated reference dataset that covers all the visual variations of the lesions. COVID-19 lesions such as GGO, crazy paving, air-space consolidation, bronchovascular thickening, and traction bronchiectasis are highly variable and have complex appearances. They differ in size, texture, shape, contrast, and location. Obtaining a rich manually annotated dataset is again a cause of concern as labeling a large number of CT images is time consuming, expensive, and highly dependent on the perspective and knowledge of the expert. To overcome these issues, few authors have used the HITL strategy for delineation. Even though this can accelerate the process, the annotations may be more inclined toward the algorithm results and can produce weak and noisy labels.

The implementation of these models for developing a clinical workstation for COVID-19 diagnosis requires appropriate hardware resources. Further, the application of CNN models in clinical practice requires careful assessment and confirmation from clinical experts regarding their usefulness in solving the issue. This is beyond the mathematical precision and accuracy of the model. The proposed CNN models should be able to understand and apply the right epidemiologic principles for disease surveillance and investigation activities. However, only a few researchers have used clinical data and conducted experiments under the supervision of doctors, which must be extended for the further diagnosis of COVID-19.

Researchers are consistently active in addressing the emerging challenges. The listed CNN models are still in the development stage, and their utility in COVID-19 diagnosis is unproven. The expectation is that promising outcomes can be accomplished using the proposed CNN models. The availability of the standard dataset along with the ground truth can help in the design of better models for the diagnosis and prognosis of COVID-19. A dedicated medical pretrained network can presumably be proposed once such a dataset becomes accessible, which may further encourage deep learning research on COVID-19. These models are plausible tools to manage and combat current and future public health emergencies in a more systematic, economic, and timely manner. While considering the pandemic, these tools can help bridge the gap between different fields and facilitate the discovery of a solution.

## Conclusions

In this article, we presented a comprehensive review of various studies conducted using CNNs to effectively detect and diagnose COVID-19 patients. The CNN models listed performed the detection, classification, and segmentation of COVID-19 images. Transfer learning facilitated the utilization of pretrained neural network models for accomplishing the different tasks. Most of the models used smaller datasets. The lack of publicly available datasets hinders the use of large medical images for training. Despite these challenges, CNN models exhibit good performance accuracy.

The experimental study conducted using a CNN framework for the classification of chest CT images into COVID-19 and non-COVID-19 cases showed an accuracy of 93% and an F1-score of 0.93. The experiment was conducted using a small dataset, without data augmentation. Moreover, the training epoch value was maintained at a small value to avoid overfitting. The proposed CNN model was trained and tested only on 746 CT images, for which good accuracy was achieved. The model must be trained and tested with more samples to result in increased generalization, which will further improve the accuracy and robustness. Nevertheless, the study conducted showed that CNN can effectively contribute to the development of a COVID-19 automated diagnostic system. A well-defined dataset along with clinical supervision can help develop CNN models for implementation in real time.

## Data Availability

The dataset analyzed during the current study is publicly available (https://github.com/UCSD-AI4H/COVID-CT).

## Abbreviations

COVID-19: Coronavirus disease
CT: Computed tomography
ReLu: Rectified linear unit
VGG19: Visual Geometry Group Network with 19-layered CNN
ResNet-V2: Residual network V2
CNN: Convolutional neural network
RT-PCR: Reverse transcription-polymerase chain reaction
GAN: Generative adversarial network
GGO: Ground-glass opacity
DeTraC: Decompose, transfer, and compose
VGG: Visual geometry group network
IRRCNN: Inception Residual Recurrent Convolutional Neural Network
HITL: Human in the loop
COPLE-Net: COVID-19 pneumonia lesion segmentation network
VB-Net: Voxel-based broad learning network
